# Evaluation of strain averaging area and strain estimation errors in a spheroidal left ventricular model using synthetic image data and speckle tracking

**DOI:** 10.1186/s12880-021-00635-y

**Published:** 2021-06-30

**Authors:** Jakub Żmigrodzki, Szymon Cygan, Krzysztof Kałużyński

**Affiliations:** Faculty of Mechatronics, Institute of Metrology and Biomedical Engineering, Warsaw University of Technology, Warsaw, Poland

**Keywords:** Block matching, Echocardiography, Averaging area, Speckle tracking echocardiography, Strain imaging, Synthetic ultrasonic data

## Abstract

**Background:**

In majority of studies on speckle tracking echocardiography (STE) the strain estimates are averaged over large areas of the left ventricle. This may impair the diagnostic capability of the STE in the case of e.g. local changes of the cardiac contractility. This work attempts to evaluate, how far one can reduce the averaging area, without sacrificing the estimation accuracy that could be important from the clinical point of view.

**Methods:**

Synthetic radio frequency (RF) data of a spheroidal left ventricular (LV) model were generated using FIELD II package and meshes obtained from finite element method (FEM) simulation. The apical two chamber (A2C) view and the mid parasternal short axis view (pSAXM) were simulated. The sector encompassed the entire cross-section (full view) of the LV model or its part (partial view). The wall segments obtained according to the American Heart Association (AHA17) were divided into subsegments of area decreasing down to 3 mm^2^. Longitudinal, circumferential and radial strain estimates, obtained using a hierarchical block-matching method, were averaged over these subsegments. Estimation accuracy was assessed using several error measures, making most use of the prediction of the maximal relative error of the strain estimate obtained using the FEM derived reference. Three limits of this predicted maximal error were studied, namely 16.7%, 33% and 66%. The smallest averaging area resulting in the strain estimation error below one of these limits was considered the smallest allowable averaging area (SAAA) of the strain estimation.

**Results:**

In all AHA17 segments, using the A2C projection, the SAAA ensuring maximal longitudinal strain estimates error below 33% was below 3 mm^2^, except for the segment no 17 where it was above 278 mm^2^. The SAAA ensuring maximal circumferential strain estimates error below 33% depended on the AHA17 segment position within the imaging sector and view type and ranged from below 3–287 mm^2^. The SAAA ensuring maximal radial strain estimates error below 33% obtained in the pSAXM projection was not less than 287 mm^2^. The SAAA values obtained using other maximal error limits differ from SAAA values observed for the 33% error limit only in limited number of cases. SAAA decreased when using maximal error limit equal to 66% in these cases. The use of the partial view (narrow sector) resulted in a decrease of the SAAA.

**Conclusions:**

The SAAA varies strongly between strain components. In a vast part of the LV model wall in the A2C view the longitudinal strain could be estimated using SAAA below 3 mm^2^, which is smaller than the averaging area currently used in clinic, thus with a higher resolution. The SAAA of the circumferential strain estimation strongly depends on the position of the region of interest and the parameters of the acquisition. The SAAA of the radial strain estimation takes the highest values. The use of a narrow sector could increase diagnostic capabilities of 2D STE.

## Introduction

Speckle tracking echocardiography (STE) is a relatively recent noninvasive technique that enables assessment of deformation within the cardiac muscle. The information obtained in this way is quantitative, which allows objective assessment of the cardiac contractility. The diagnostic potential of the STE seems significant however the method is not a routine element of the current cardiac diagnostic.

In majority of published studies and in implementations available on ultrasonic scanners the displacement and strain measures of the left ventricle are averaged over large areas, most frequently over the entire wall visible in the projection used or over large segments defined accordingly to some standard, e.g. American Heart Association AHA17 [1]. The limited spatial resolution of the STE may impair its diagnostic capability in the case of diseases resulting in local changes of the cardiac contractility (e.g. non-transmural infarction). The knowledge on the resolution of the STE is limited and not systematized. There are results indirectly indicating, that the cardiac deformation measures may be estimated with higher resolution than it is currently done [2–5]. Quantitative assessment of resolution is made by Chakraborty et al. [6] and Tabassian et al. [5]. The first work attempts to identify in an “in silico” study the area of the left ventricular (LV) model featuring compromised contractility and claims that area of at least 1.9 cm diameter may be detected. The second work attempts to evaluate clinical utility of the STE using different spatial resolutions.

It is of interest to address the resolution of the STE together with accuracy of the strain estimation, as both are important from the clinical point of view. There were numerous studies on the STE accuracy [7–20], however, comparison of their results is difficult, due to different study design or different error measures used. One of the conclusions of these works is that the accuracy of global strain estimates is of order of several hundredths and that of global displacement estimates is about 1–2 mm. Some results indicate a link between estimation accuracy and the direction of the displacement with respect to the ultrasonic wave propagation direction [2, 7, 11, 14, 20]. The errors of the estimate of the deformation perpendicular to the wave propagation direction are usually greater than those of the deformation parallel to this direction. There is also a link between the frame rate, line density and estimation accuracy [21–23], in part explained by the relation between tissue deformation and data decorrelation [24–26]. Studies, where the strain estimation was carried out with high accuracy, usually present results qualitatively (maps), whereas quantitative measures are usually averaged over the entire wall [2, 8, 11, 14].

This work attempts to evaluate, how far one can reduce the strain averaging area, thus increase the resolution, which may be of value in the case of disease-related local abnormalities such as cardiac ischemia. An important issue in this case is to decide when this area reduction becomes no longer reasonable or justified. This may be based on the rationale that the reduction of this area should not result in strain estimation errors high enough to impair the distinction between the viable cardiac tissue and the ischemic one.

It is not possible to propose one particular value of the largest allowable strain estimation error as it depends on the application. In the majority of clinical studies using STE a single, empirical threshold for differentiating particular dysfunctions is sought for [27]. Inability to provide a perfect differentiation method (area under the ROC curve is < 1) results from two main sources. First that strain values in myocardium, both healthy and dysfunctional, seem to have a wide distribution, second that the measurement method itself is uncertain. Available STE methods use strain averaging over a relatively large, arbitrary area. This area can encompass both types of tissue with unknown proportions, thus the resulting strain value is a weighted average of unknown values with unknown weights.

Available data allows for an assumption that the viable tissue shows layer-specific longitudinal strain lower than − 0.12 [27, 28]. Studies attempting to assess local strains directly in dysfunctional tissue are scarce. Work presented in [4] reports that the non-viable transmural scar tissue in ischemic heart disease shows longitudinal strain values greater than − 0.05.

In this study we address the trade-off between the strain averaging area and estimation error and how it depends on selected imaging parameters. We search for the smallest averaging area (i.e. SAAA) for which estimation error does not exceed an assumed limit. Proposed here error limits are based on restricted available clinical data [4, 27, 28] and should be updated when new results are reported.

The results of this study may be of interest from the point of view of diagnosis of local changes of the cardiac contractility. A Finite Element Method (FEM) reference model and synthetic ultrasonic data based on this model are used in this study, as in all other situations (clinic, animal studies, physical models) a reliable, high resolution and concurrent reference data is difficult to obtain. Although more advanced and more realistic synthetic datasets are publicly available, such as a database published in [29], based on an numeric electromechanical model of the heart and using real life echocardiographic images to form synthetic ones, we have decided to use our own, relatively simple synthetic data, to be able to freely set different parameters of ultrasonic data acquisition like projection planes, number of lines and frame rate which are fixed in other data.

## Material and methods

### Mechanical model of the left ventricle

**Fig. 1:**
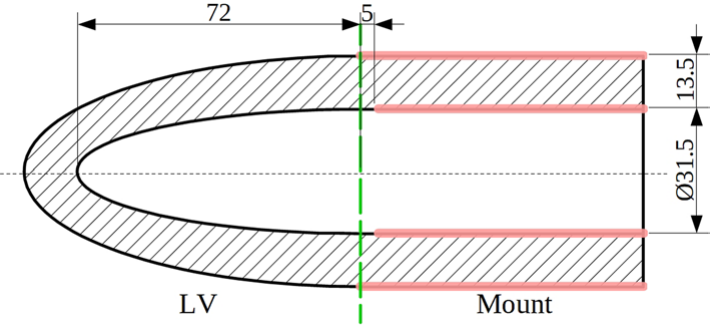
Cross-section of LV model with mounting part at ES state. Mounting surfaces marked by red lines. Dimensions in millimeters

The LV model exploited in this study was based on a physical LV phantom [30], similarly as presented previously [2, 7, 31, 32]. Modeling technique and all the properties of the model resulting from extensive studies were described in [33]. In the current work the 3D model was reduced in size compared to those used previously and had a shape of a half spheroid at the ES phase (Fig. 1). The semi-axes of internal surface of the model at ES phase were 72 mm and 15.75 mm. Semi-axes of the external surface of the model at ES were 85.5 mm and 29.25 mm. The model was extended at the base by a cylindrical element which was used to immobilize the model during FEM simulation of deformation (Fig. 1). The active LV part of the model was divided into 13,740 hexahedral elements defined by 16,950 nodes. The constraints were applied to the entire external surface of this cylinder and on its internal surface except for a 5 mm strip nearest to the ventricle model, in order to reduce excessive strains at the inner border of the cylinder and the half spheroid. Deformation of the LV model was simulated using Abaqus 6.13–3 (Dassault Systèmes Simulia Corp, Providence, RI, USA) FEM software.

The material of the model was defined as hyperelastic with Poisson’s ratio of 0.45, since this material model has been proven to best resemble the material used for physical LV phantoms [30, 31]. As a result of those previous studies, Yeoh constitutive hyperelastic material model was used to describe its behavior in FEM simulations. Coefficients of the model were calculated using the Abaqus software based on the provided stress–strain curve obtained during the uniaxial deformation testing of polyvinyl alcohol (PVA) cryogel samples, as this is the material frequently used for physical phantom construction. This strain–stress relationship can be empirically described by the function

1 $$\sigma = 7525.8 \cdot e^{{10.25 \cdot \varepsilon }} - 7525.8,$$

where *ε*—strain [1], *σ*—stress (Pa).

**Fig. 2:**
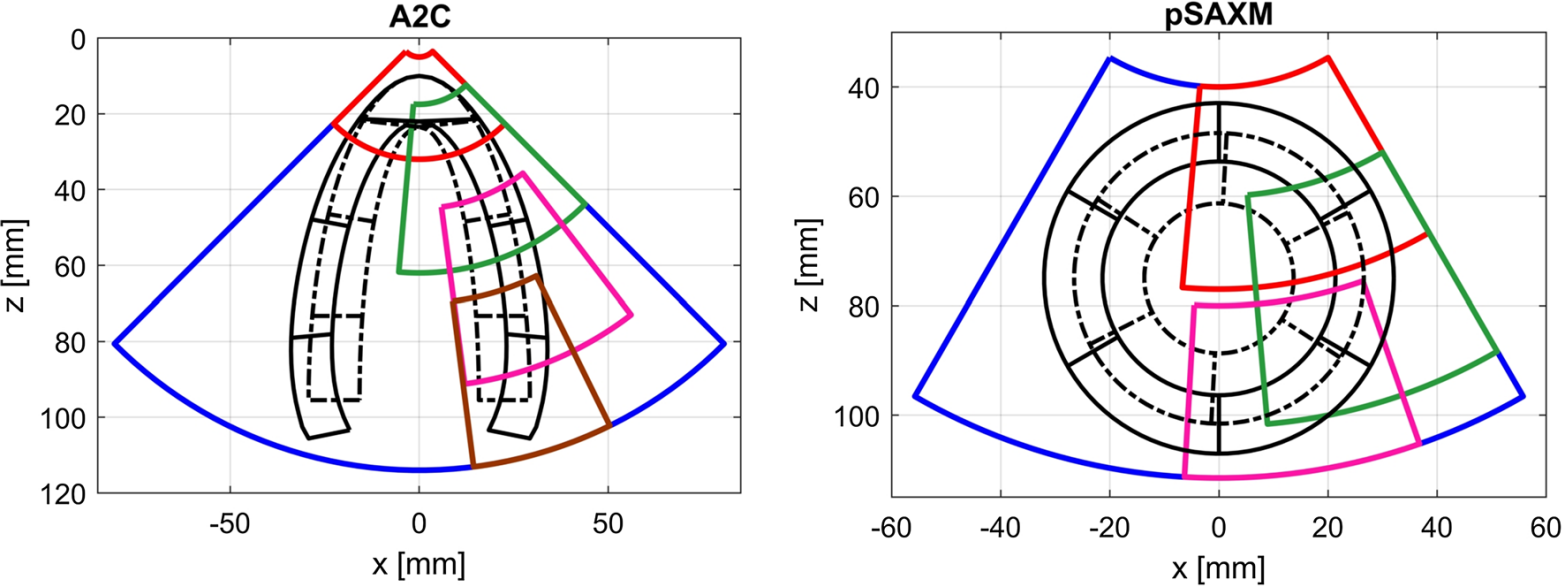
Normalized amplitude of pressure load and rotation used during simulation of the LV model deformation

Deformation was forced by application of the time-varying pressure (Fig. 2) to the inner surface of the model (Fig. 1). The peak pressure was set to 30 kPa and resulted in a deformation close to the one observed clinically (Table 1).

**Table 1:** Shape and deformation parameters of the LV model

Parameter	Model	Reference range	
		Min	Max
LVEDd (mm)	46	40	50
LVESd (mm)	31	20	40
LVEDv (cm^3^)	94.5	50	150
LVESv (cm^3^)	37.4	12	60
AVPD (mm)	− 10.1	− 5.2	− 20.4
Apical rotation (°)	13	6.5	19.5
Basal rotation (°)	− 6.9	− 3.4	− 10.4
GRS (%)	20.3	17	67
GCS (%)	− 26.5	− 14	− 28
GLS (%)	− 13.6	− 12	− 25

The reference data were estimated from literature [34–37] and [38–47], respectively. *LVEDd* Left ventricular end-diastolic diameter; *LVESd* Left ventricular end-systolic diameter; *LVEDv* Left ventricular end-diastolic volume; *AVPD* longitudinal atrioventricular plane displacement; *GCS* Global Circumferential Strain; *GLS* Global Longitudinal Strain; *GRS* Global Radial Strain

The rotation of the LV was added to data obtained from the FEM simulation as an additional angular displacement of the FEM mesh vertices. The long axis (LAX) of the model constituted the axis of the rotation. The direction and value of the angular displacement were defined as [48]:

2 $$\theta \left( {P,t} \right) = \frac{{\theta _{B} \left( t \right) - \theta _{A} \left( t \right)}}{{\frac{2}{3}LA\left( t \right)}} \cdot \left( {x_{P} \left( t \right) - \frac{1}{6}LA\left( t \right)} \right) - \theta _{B} \left( t \right),$$

3 $$\theta _{B} \left( t \right) = \theta _{{B\_ES}} \cdot {\text{Norm}}\left( t \right),$$

4 $$\theta _{A} \left( t \right) = \theta _{{A\_ES}} \cdot {\text{Norm}}\left( t \right),$$

where *Θ*(*P,t*)—the angular displacement of the point *P* at the time instant *t*; *LA*(*t*)—total length of the LAX at the time *t*; *x*_*p*_(*t*)—the distance between the projection of the vertex *P* at LAX and the intersection of LAX and the internal surface of LV model (e.g. apex) at the time *t*; *Θ*_*A ES*_—the peak value of rotation at the apical level, equals 13° [42], *Θ*_*B ES*_—the peak value of rotation at the basal level equals − 6.9° [42], Norm(*t*)—normalized function of rotation (Fig. 2).

As the base of the model was immobilized during the simulation, the maximal displacement along the LAX direction occurred at the apex of the model, contrary to the clinical observation [49]. Correction of the displacement distribution along the LAX was made by subtracting from all the displacement vectors of the FEM mesh vertices the mean displacement in the LAX direction estimated at the distance of 0.2 of the LAX length from the apex. The value of the displacement along LAX is then expected to be close to zero at this section [49].

### Synthetic ultrasonic data

**Fig. 3:**
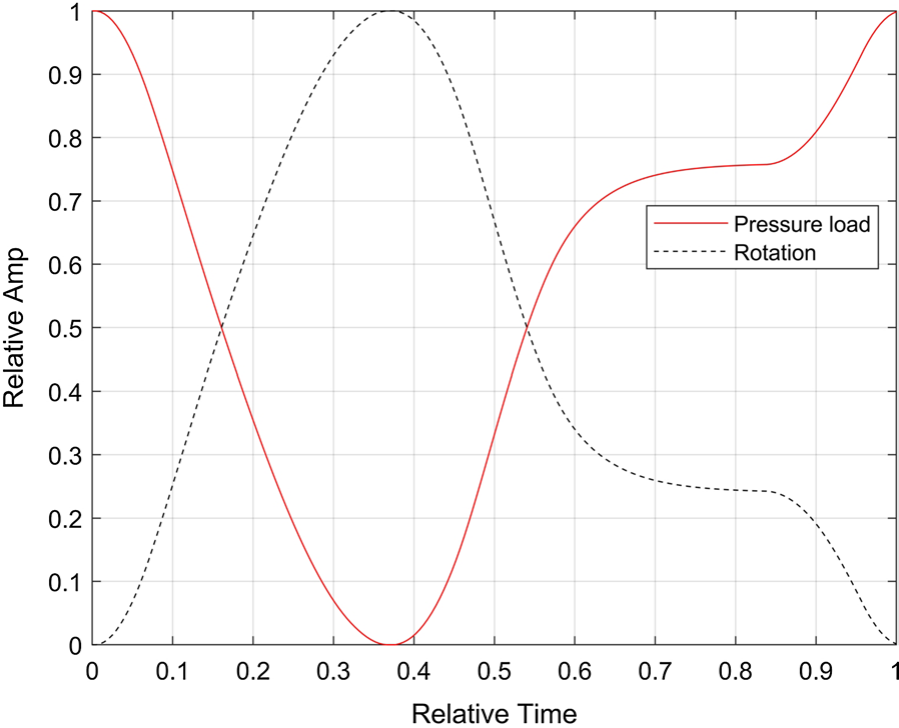
Position and size of all image sectors used in the A2C (left) and pSAXM (right) projections. The black lines denote position and shape of the visible LV model wall at ES (dashed line) and ED (solid line). The views are numbered according to the Table 2: 1—blue line (right), 2—red line (right), 3—green line (right), 4—magenta line (right), 5—blue line (left), 6—red line (left), 7—green line (left), 8—magenta line (left), 9—brown line (left)

Synthetic RF data were generated analogically as in [7]. Briefly, the RF data were simulated using FIELD II package [50, 51] and meshes obtained from the FEM simulations. Two imaging projections were simulated, i.e. the apical two chamber (A2C) view and the mid parasternal short axis view (pSAXM). The A2C imaging plane was set along the LAX of the LV model. The pSAXM imaging plane was perpendicular to the LAX of the LV model and located 5.2 cm above the apex at the end-diastolic phase. The sector position and size were adjusted to encompass the entire cross-section of the LV model (full view) or, alternatively, only its part (partial view), covering in each case one segment of the 17-segment left ventricle segmentation scheme (AHA17). The positions and sizes of all image sectors are shown in the Fig. 3. The parameters of image acquisition are presented in the Table 2. 10 instances of the synthetic image data were generated for each view with random positions of scatterers, resulting in different speckle patterns for each data set.

**Table 2:** Image acquisition parameters

View nos	Projection	Imaged AHA17 segments	Image sector parameters				Number of lines	Focus depth (cm)	FPS (1/s)	View type
			D_min_ (cm)	D_max_ (cm)	T (°)	W (°)				
1	pSAXM	7–12	4	11.15	0	60	106	7.575	65	Full
2		7	4	7.7	− 12.5	35	153	5.85	65	Part
3		12	6	10.2	− 17.5	25	116	8.1	65	Part
4		11	8	11.15	− 8	22.5	106	9.575	65	Part
5	A2C	1, 4, 7, 10, 13, 15, 17	0.5	11.4	0	90	103	5.95	65	Full
6		17	0.5	3.2	0	90	370	1.85	65	Part
7		13	1.75	6.2	− 20	50	191	3.975	65	Part
8		7	4.5	9.2	− 22.5	30	128	6.85	65	Part
9		1	7	11.4	− 16.75	19	103	9.2	65	Part

Notation: D_min_—depth of sector beginning; D_max_—sector end depth; T—sector tilt; W—sector width; FPS—frame per second

### Strain estimation

The estimation of the spatial distribution of strain was split into 3 main steps, i.e. preprocessing of the synthetic image data, estimation of the displacement over the entire deformation cycle, estimation of strain components in anatomical directions.

The masks used for the segmentation of the synthetic images were obtained from reference data derived from the FEM simulation to limit the number of factors affecting the results of the experiment.

The anatomical directions where identified in all points of the LV model wall cross-section using the abovementioned masks. In the case of image data obtained using the pSAXM projection the radial direction was defined as the direction of the vector connecting chosen point of LV model wall and the point of intersection of the LAX with imaging plane. This intersection point position was identified using the FEM data. The circumferential direction in each point of the LV model wall cross-section was obtained as orthogonal to the radial direction. In the A2C projection the inner and outer border of the LV model wall cross-section were detected using the segmentation masks. Then, the longitudinal direction in border points was found as the direction of the vector tangential to the border. Finally, longitudinal direction was linearly interpolated at all points of the LV model wall cross-section. The radial direction was found as the direction orthogonal to the longitudinal.

**Fig. 4:**
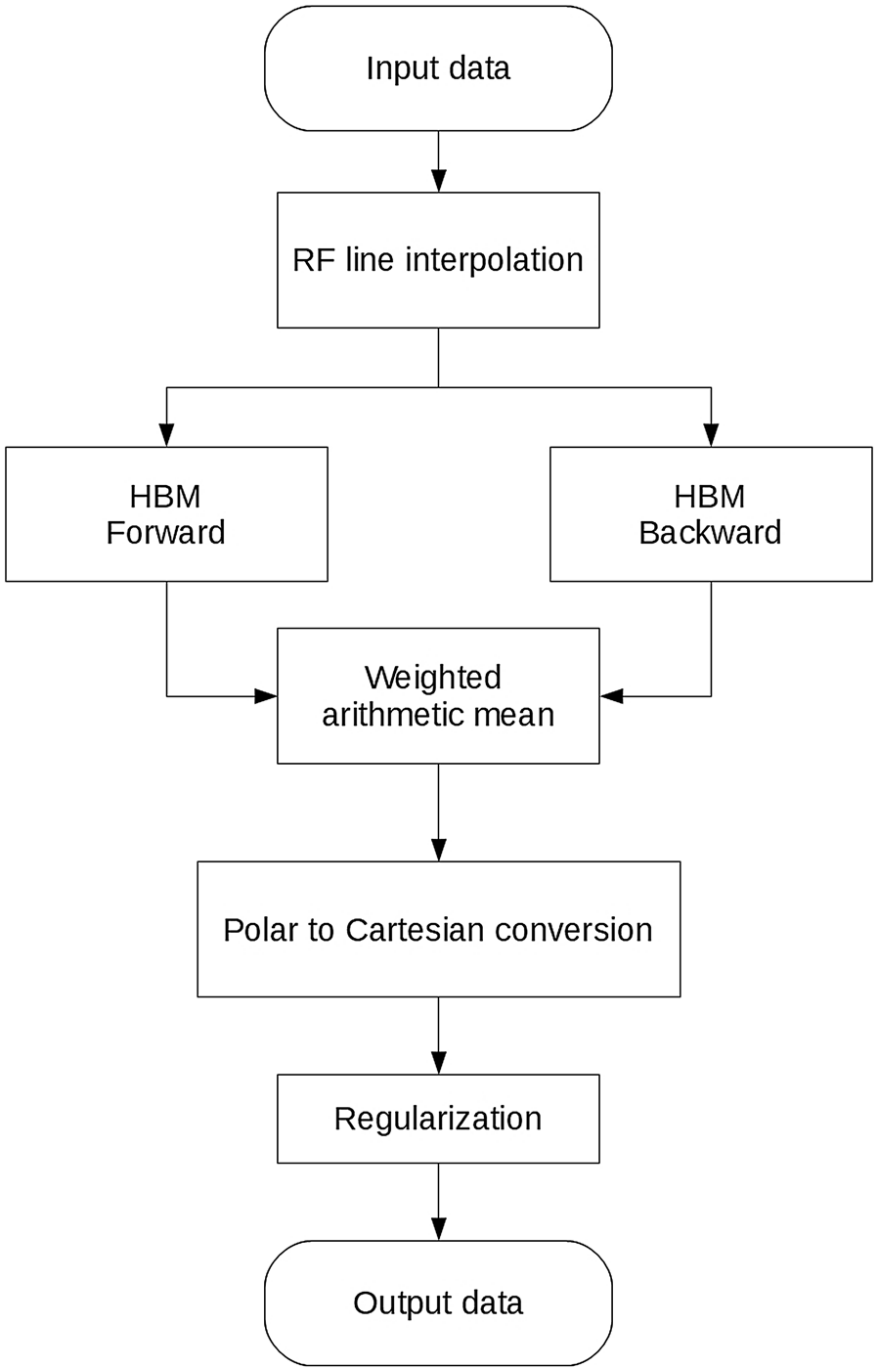
Main algorithm of interframe displacement estimation

Incremental (interframe) displacements were estimated using a hierarchical block matching method (HBM) [52] in implementation similar to that described in [11], expanded by two-directional incremental displacement estimation [2, 7, 32]. The main algorithm is shown in the Fig. 4. 65 frames of RF signals registered during one simulated cardiac cycle and the corresponding LV model wall masks constituted the input data. The incremental displacement was estimated in polar coordinate system with its origin in the center of the ultrasonic transducer array [7]. Firstly, RF lines were linearly interpolated 4 times. The incremental displacement estimation in both directions was carried out in three iterations. The parameters of each iteration are specified in the Tables 3 and 4. The dimensions of the kernel window in the first iteration were set to 15-fold multiple of the maximal axial and lateral displacement, obtained from reference data. The dimensions of the search window in the first iteration were set to 18-fold multiple of the maximal displacements. The number of iterations and multiplication coefficients were chosen to minimize the estimation errors and computation time. The dimensions of both windows were reduced 4 times in each further step. These dimensions fulfilled additional constraints as follows:

1. The size of the kernel was at least 3 wavelengths and 3 lines in axial and lateral direction respectively.
2. The dimension of the search window exceeded that of the kernel by at least four RF signal samples in the axial direction and four lines in the lateral direction.
3. Window dimension was rounded to the nearest integer number of RF signal samples or lines.

**Table 3:** Parameters of the HBM method

Parameter	Iteration number		
	1	2	3
Similarity measure	Cross-correlation coefficient		
Input signal	RF envelope	RF envelope	RF
Kernel overlap	2/3		
Method to obtain subpixel resolution	Similarity measure map interpolation using cubic B-spline		
Subpixel resolution	16x	32x	64x

**Table 4:** Kernel and search window dimensions at different stages of the HBM algorithm

View no	Kernel window						Search window					
	Iteration 1		Iteration 2		Iteration 3		Iteration 1		Iteration 2		Iteration 3	
	A (mm)	L (°)	A (mm)	L (°)	A (mm)	L (°)	A (mm)	L (°)	A (mm)	L (°)	A (mm)	L (°)
1	11.59	10.69	2.89	2.71	0.81	0.71	13.90	12.97	3.50	3.28	1.04	1.28
2	11.59	10.40	2.89	2.59	0.81	0.63	13.90	12.47	3.50	3.16	1.04	0.86
3	11.59	10.57	2.89	2.66	0.81	0.70	13.90	12.74	3.50	3.20	1.04	0.92
4	11.59	10.42	2.89	2.62	0.81	0.69	13.90	12.56	3.50	3.15	1.04	0.91
5	11.59	13.42	2.89	3.30	0.81	1.10	13.90	16.06	3.50	4.18	1.04	1.98
6	11.59	11.03	2.89	2.74	0.81	0.67	13.90	13.22	3.50	3.35	1.04	0.91
7	11.59	10.84	2.89	2.69	0.81	0.72	13.90	13.07	3.50	3.35	1.04	0.99
8	11.59	10.67	2.89	2.65	0.81	0.65	13.90	12.79	3.50	3.24	1.04	0.88
9	11.59	10.45	2.89	2.65	0.81	0.70	13.90	12.59	3.50	3.21	1.04	0.88

*A—*axial dimension, *L—*lateral dimension

The incremental displacement was estimated for each pair of consecutive frames as the weighted average of estimates calculated in forward direction (between frame with index *n* and *n* + 1) and in backward direction (between frame with index *n* + 1 and *n*). The values of the cross-correlation coefficient calculated for each point of the LV model wall in both directions were used as weights. The maps of the estimated incremental displacement components were converted to Cartesian coordinates and interpolated over a 0.2 mm regular grid. Next step was the regularization of the incremental displacement maps using the cubic b-spline method [53, 54]. The regularization was implemented using the Matlab function ‘csaps’. The regularization coefficient was experimentally set to (1–10^−6.5^). Regularized estimates of the incremental displacement components constituted output data of the interframe displacement estimation process.

The interframe displacement accumulation procedure was carried out as previously described [7]. The incremental displacements were interpolated outside the LV model wall using gaps filling method [55, 56] to reduce errors of displacement accumulation in the vicinity of the model borders. Subsequently the accumulation of incremental displacements was carried out for each point within the model wall accordingly to the evolution of the deformation process and in the opposite direction. The obtained displacements curves along the deformation cycle were weight averaged [7, 57, 58].

The values of the components of the Lagrangian finite-strain matrix were estimated similarly as described previously [7]. The only difference was that the directional derivatives of displacements were computed analytically using cubic b-spline interpolant function fitted to the estimated displacement fields. The coefficients of the interpolant function were obtained using the Matlab function ‘csapi’. The strain matrix components (longitudinal, radial, and circumferential) in anatomical directions were obtained as described in [7, 8].

**Fig. 5:**
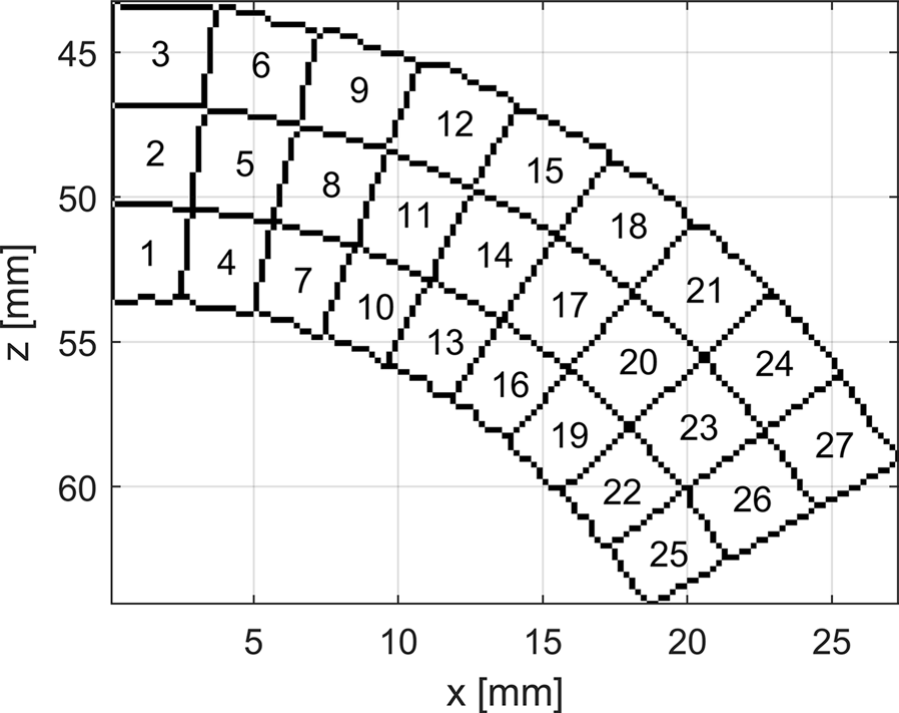
Division of the AHA17 segment no 7 into subsegments with N_LC_ = 9 in the longitudinal direction and N_R_ = 3 in the radial direction. Subsegment indices (SSN) are specified in subsegments. pSAXM projection

**Fig. 6:**
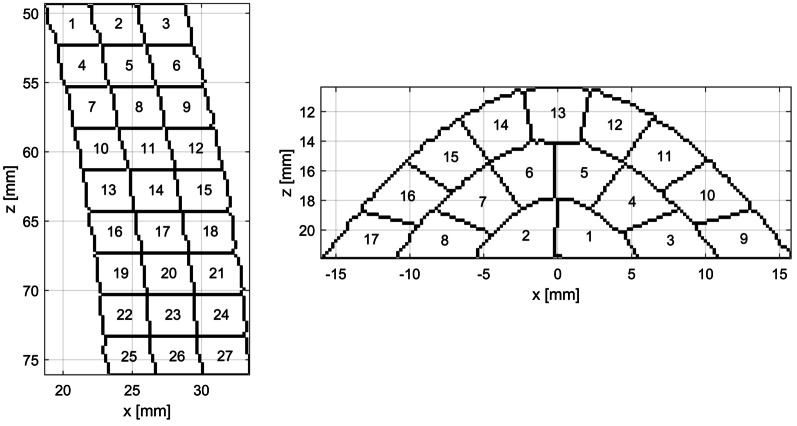
Division of the AHA17 segment no 7 (left) and 17 (right) into subsegments with N_LC_ = 9 in the longitudinal direction and N_R_ = 3 in the radial direction. Subsegment indices (SSN) are specified in subsegments. A2C projection

**Fig. 7:**
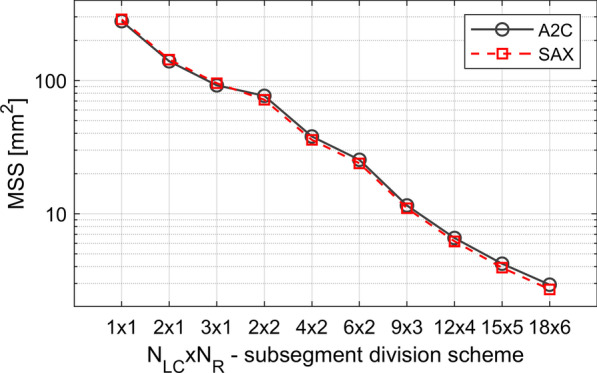
MSS of AHA17 segments viewed in the A2C (black solid line) and pSAXM (red, dashed line) projection measured for different subsegment division scheme (Table 5). MSS—mean value of the subsegment surface. N_LC—_number of subsegments in longitudinal or circumferential direction. N_R—_number of subsegments in radial direction

The estimated strain components maps were divided into AHA17 segments. The mean area of the AHA17 segment as applied to the model used here equals approximately 280 mm^2^ (Fig. 7). The AHA17 segments were divided into subsegments. 10 division schemes were applied (Table 5). The AHA17 segments imaged in the pSAXM projection were divided into N_LC_ circumferential subsegments and N_R_ radial subsegments (Table 5; Fig. 5). All AHA17 segments of the A2C projection, except the apical segment no 17, were divided into N_LC_ longitudinal subsegments and N_R_ radial subsegments (Table 5; Fig. 6 left part). The 17-th segment was divided radially into N_R_ layers, then the outermost layer was divided into N_LC_ subsegments along longitudinal direction. Other radial layers were divided longitudinally into subsegments with areas equal to that of the subsegment in the outermost layer (Table 5; Fig. 6, right part). The mean value of the subsegment surface area (MSS) as a function of the division scheme is shown in the Fig. 7. The strain estimates were averaged over the segment/subsegment surface for each frame of the image sequence.

**Table 5:** AHA17 segment division scheme

Division scheme No	1	2	3	4	5	6	7	8	9	10
*N*_*LC*_	1	2	3	2	4	6	9	12	15	18
*N*_*R*_	1	1	1	2	2	2	3	4	5	6

N_LC_—number of subsegments in longitudinal or circumferential direction. N_R_—number of subsegments in radial direction

Values of N_LC_ and N_R_ applied in division of the segments were chosen to preserve as close as possible the proportion of the longitudinal/circumferential dimension to the radial dimensions of the subsegments resulting from each division and to obtain monotonic decrease of the MSS.

## Reference data

The reference values of interframe displacement were calculated by linear interpolation of the displacements of the FEM mesh vertices to the synthetic RF data resolution. The reference values of strains were estimated analogously as the estimates obtained from synthetic image data. The only difference was the value of the regularization coefficient which was set to (1–10^−9^). This value was chosen experimentally as the minimal value which ensured correction of the artefacts of the displacements maps resulting from the finite size of the FEM mesh elements.

### Error metrics

To assess the errors of the strain estimates three metrics were used, namely a measure of the end-systolic strain estimation error *E*_*ES*_ (5); a measure of the end-systolic strain estimation relative error *NE*_*ES*_ (6), and the cross-correlation coefficient *XCC* (7) between the curve of the estimate over the deformation cycle and the corresponding reference plot:

5 $$E_{{ES}} \left( x \right) = x_{E} \left( {t_{{ES}} } \right) - x_{R} \left( {t_{{ES}} } \right),$$

6 $$NE_{{ES}} \left( x \right) = \frac{{E_{{ES}} \left( x \right)}}{{x_{R} \left( {t_{{ES}} } \right)}} \cdot 100\% ,$$

7 $$XCC\left( x \right) = \frac{{\mathop \sum \nolimits_{{t = 1}}^{T} (x_{E} \left( t \right) - \overline{{x_{E} }} )(x_{R} \left( t \right) - \overline{{x_{R} }} )}}{{\sqrt {\mathop \sum \nolimits_{{t = 1}}^{T} (x_{E} \left( t \right) - \overline{{x_{E} }} )^{2} } \sqrt {\mathop \sum \nolimits_{{t = 1}}^{T} (x_{R} \left( t \right) - \overline{{x_{R} }} )^{2} } }},$$

where *x* denotes the analyzed component of strain (*ε*_*L*_—longitudinal strain, *ε*_*C*_—circumferential strain, *ε*_*R*_—radial strain); *x*_*E*_(*t*) and *x*_*R*_(*t*)—the estimate and reference, respectively, averaged over the surface of the considered subsegment for the image with the index *t*; $\overline{{x_{E} }}$ and $\overline{{x_{R} }}$—the temporal average of *x*_*E*_(*t*) and *x*_*R*_(*t*), respectively; *t*—the index of an image in the sequence, *T*—the number of images in the sequence, *t*_*ES*_—the index of the image registered at the end-systole.

The median (8) and median absolute deviation (9) of the strain estimation error measures were used to assess the statistical properties of the estimation error:

8 $$MEDErr\left( {V,S,DS} \right) = MED\left( {Err_{{Tr = 1,~SSN = 1}} , \ldots ,~Err_{{Si = 10,~SSN = N_{{LC}} \cdot N_{R} }} } \right),$$

9 $$MADErr\left( {V,S,DS} \right) = MAD\left( {Err_{{Tr = 1,~SSN = 1}} , \ldots ,~Err_{{Si = 10,~SSN = N_{{LC}} \cdot N_{R} }} } \right),$$

where *MED*—median; *MAD*—median absolute deviation; *MEDErr*—median value of the error measure *Err* (defined in one of Eqs. 5–7); *V*—view type (projection and sector position and type, see Table 2; Fig. 3); *S*—AHA17 segment number; *DS*—division scheme (see Table 5); *N*_*LC*_—the number of subsegments in longitudinal or circumferential direction in the scheme *DS*; *N*_*R*_—the number of subsegments in radial direction in the scheme *DS*; *SSN*—the index of the subsegment; *S*_*i*_—the index of the image in the synthetic data sequence.

Finally, the value of the predicted maximal error of strain estimation was calculated as:

10 $$MaxErr = \max \left( {\left| {MEDErr - 3 \cdot MADErr} \right|,\left| {MEDErr + 3 \cdot MADErr} \right|} \right)$$

where *MaxErr*—predicted maximal value of the estimation error measure (*E*_*ES*_ or *NE*_*ES*_) of the end-systolic value of the specific strain component (longitudinal, circumferential or radial).

The use of median based measures of central tendency and dispersion is dictated by their lower sensitivity to outliers than that observed in the mean based measures [59, 60]. The standard deviation is equal to 1.4826·*MAD* for the normal distribution [60]. Assuming normal distribution of strain estimation errors it may be expected that strain estimation errors will be below *MaxErr* in 95% of cases.

It is difficult to propose an universal allowable strain estimation error, because publications presenting the values of strain estimates for viable and ischemic tissue measured with high spatial resolution are scarce, in most cases global strain measures and single strain limit are used [27]. According to available data concerning local strain estimation, the viable tissue shows layer-specific longitudinal strain smaller than − 0.12 [28], whereas the non-viable transmural scar tissue in ischemic heart disease shows longitudinal strain values greater than − 0.05 [4]. The first study is based on 119 cases, the second on 31. Using these strain values one may assume that the optimal MaxNE_ES_ limit equals 33%. In this case, assuming normal distribution of strain estimation errors, the measured strain values for viable tissue should not be greater than − 0.08, whereas strain values measured for ischemic tissue should not be lower than − 0.07 in 95% of cases. Therefore, when the error up to 33% is allowed there will be no misclassification of the viable and ischemic tissue in 95% of cases. One could therefore consider the smallest averaging area, for which the MaxNE_ES_ will not exceed 33%, as the smallest allowable averaging area (SAAA). This area may also be considered as a measure of resolution potentially useful from the clinical point of view, as for such an area the possibility of distinction between the viable tissue and ischemic tissue would be preserved. Using a higher allowable error limit, i.e. 66%, may lead to a significant increase of erroneous classification of the tissue, whereas using lower allowable error limit, i.e. 16.7%, would result in estimates obtained for large averaging areas (thus loss of resolution). Therefore this paper concentrates on data analysis and discussion of results obtained for the largest allowable error limit equal 33% and offers some comparison with data obtained for the remaining two allowable error limits. This comparison gives insight into the tradeoff between the strain estimation averaging area and accuracy of strain estimation.

The plot of MaxNE_ES_ versus MSS was analyzed. The smallest value of the MSS preceding that for which MaxNE_ES_ exceeded 16.7%, 33% or 66% was considered the SAAA, i.e. resulted in estimation error below the specific MaxNE_ES_ limit.

## Results

### LV mechanical model

**Fig. 8:**
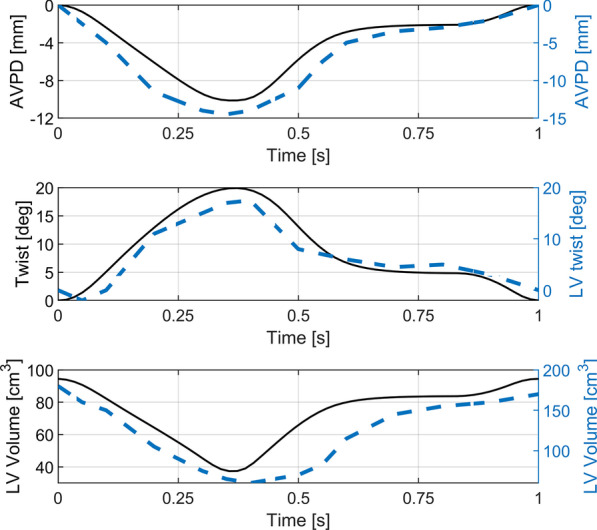
The atrioventricular plane displacement (AVPD), LV twist and LV volume change cycle. Blue dashed lines show typical plots obtained in healthy subjects, reported in [63]—AVPD, [19]—LV Twist and [64]—LV Volume

The shape of the model is spheroidal during the entire deformation cycle, accordingly to the clinical observations [61]. The largest longitudinal displacements are observed at base of the model. The LV model chamber volume, twist and longitudinal atrioventricular plane displacement (AVPD) change synchronously and reach peak values 370 ms after the end of the diastole (Fig. 8). The LV model shape and deformation parameters are within the physiological range (Table 1). The temporal evolution of the LV volume, twist and AVPD are close to those clinically observed [19, 62–64].

### Strain estimation

**Fig. 9:**
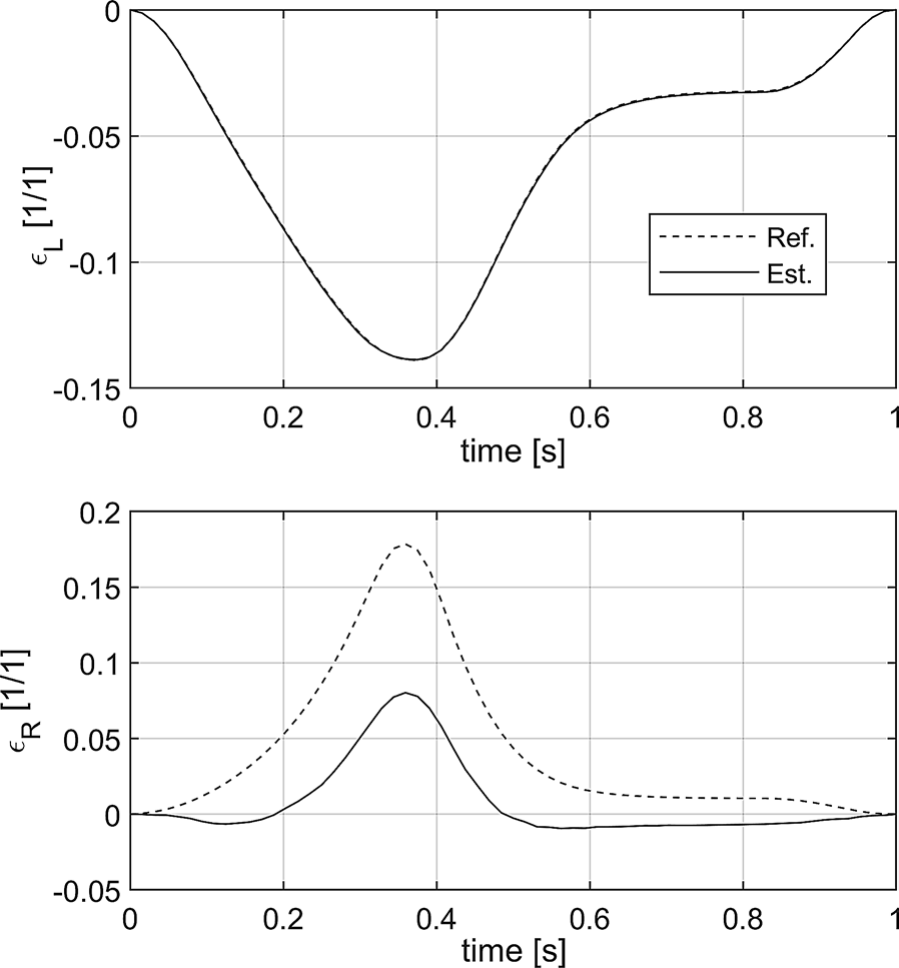
Curves of the longitudinal (ε_L_) and radial (ε_R_) strain averaged over the 7th segment of AHA17 during the simulated cardiac cycle. Data were acquired according to 8th view setting (see Table 2). Est.—strain estimates obtained in simulation. Ref.—plot obtained from MES simulation results

**Fig. 10:**
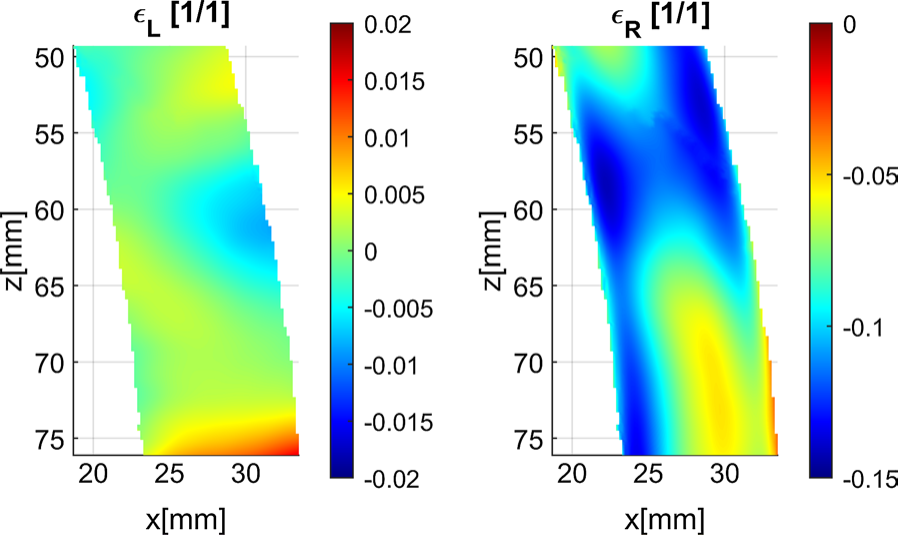
Distribution of ES strain estimation error in the 7th segment of AHA17 imaged according to 8th acquisition setting (see Table 2). Color scales are spanned differently for each map

**Fig. 11:**
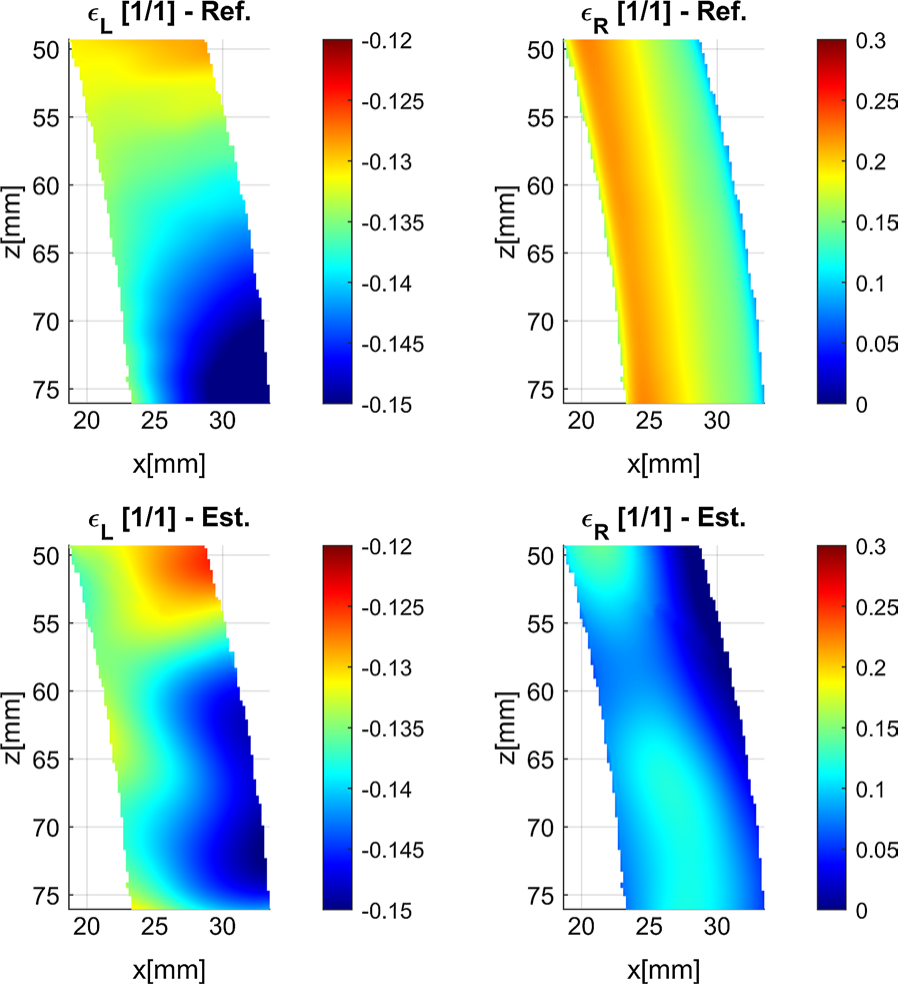
Distribution of ES strain estimate (Est.) and the corresponding reference (Ref.) in the 7th segment of AHA17 imaged according to 8th acquisition setting (see Table 2). Color scales are spanned differently for radial and longitudinal strain components

Plots of strain over the deformation cycle as well as strain maps show qualitative and quantitative difference between longitudinal and radial strain estimation accuracy (Figs. 9, 10, 11). The plot of longitudinal strain averaged over entire AHA17 segment shows almost perfect agreement with referential data, whereas that of the radial strain estimate shows high relative error (close to 50%), as well as negative values of strain at the beginning and at the end of the cycle. Spatial maps of ES strain error show that longitudinal strain error is symmetrically distributed around zero, whereas the radial strain error ranges from − 0.04 to − 0.15 and indicates strong bias of the strain estimate. The maps of the ES strain estimates and corresponding reference distributions show considerable similarity in the case of the longitudinal strain (Fig. 11). In the case of the radial strain estimate map only moderate correlation to referential distribution and a strong bias are observed, despite similar to reference data relatively high spatial gradient of strain.

**Fig. 12:**
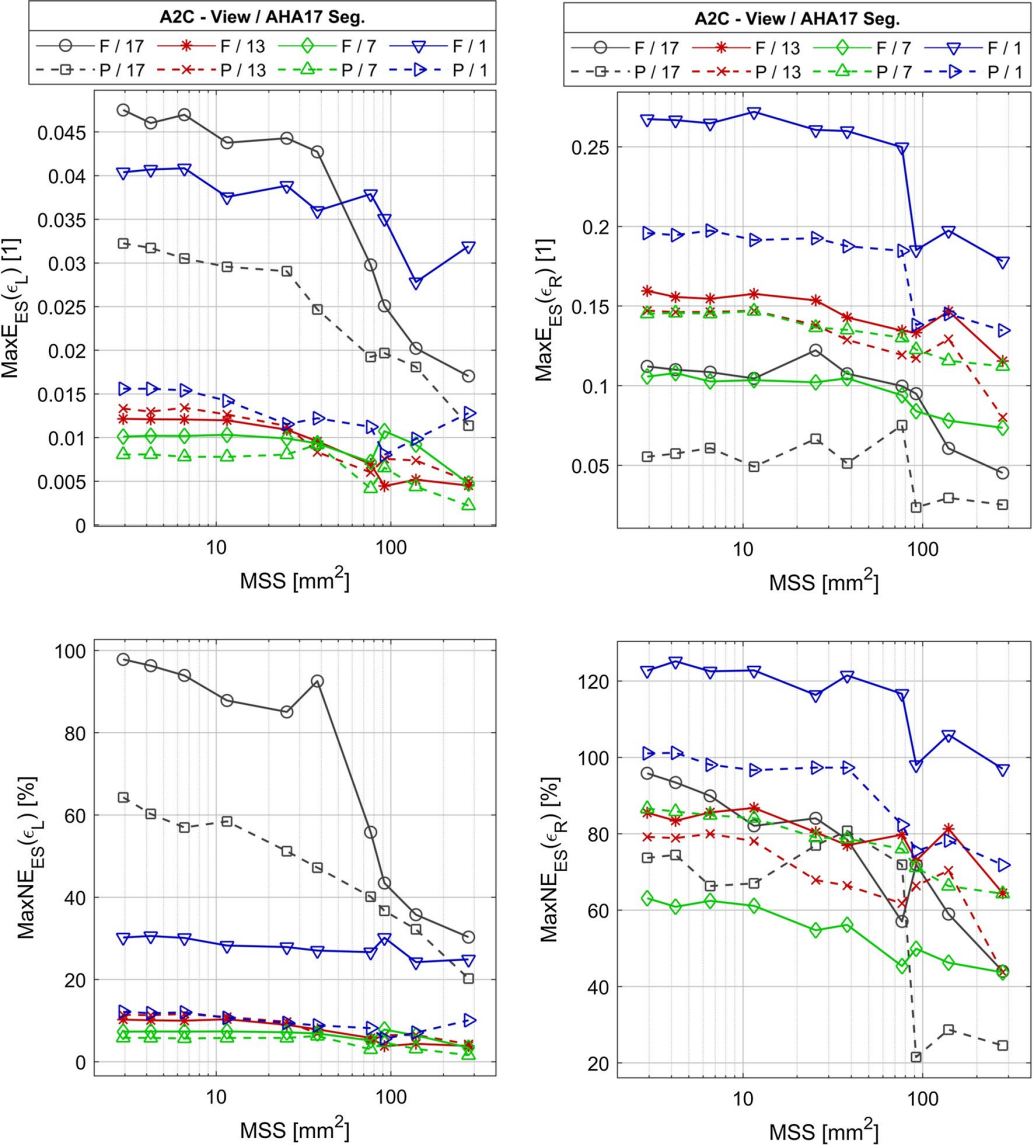
Plots of maximal absolute error (MaxE_ES_) and maximal normalized error (MaxNE_ES_) of longitudinal (ε_L_) and radial (ε_R_) as a function of mean surface area of subsegments viewed in the A2C projection. *F* full view; *P* partial view. *MSS* mean value of the subsegment surface

The values of *MaxE*_*ES*_ and maximal normalized (*MaxNE*_*ES*_) error of longitudinal strain estimates (*ε*_*L*_) are in range from 0.002 to 0.048 and from 1.6 to 98% respectively (Fig. 12 left). Both errors tend to decrease as the size of subsegment increases. These errors have lower or similar values if partial views are used as compared to the results for the full view, and particularly large difference is observed for the AHA17 segments no 1 and 17. The smallest values of errors are measured for the AHA17 segment no 7 imaged using a partial view. The values of *MaxE*_*ES*_ and *MaxNE*_*ES*_ in this case do not exceed 0.009 and 6% respectively. The *SAAA* values measured for the *MaxNE*_*ES*_ limit of 33% are below 3 mm^2^ regardless of the view type in all segments, except for the segment no 17. The *SAAA* values obtained for this segment are 278 mm^2^ for the full view and 139 mm^2^ for the partial view what corresponds to the case of no division or division into 2 subsegments, respectively (Table 6). There are few differences in *SAAA* values for other *MaxNE*_*ES*_ limits (Table 6). The increase of the *SAAA* is observed for the *MaxNE*_*ES*_ limit of 16.7% in segments 17 (full and partial view) and 1 (full view only). In other cases, *SAAA* is below 3 mm^2^ regardless of the *MaxNE*_*ES*_ limit. The *MXCC* values exceed 0.998 in all AHA17 segments viewed in the A2C projection regardless of used view type for the *SAAA* obtained when the MaxNE_ES_ limit equals 33% (Table 7). For all segments in the A2C projection the value of *MED* and *MEDN* is in the range from − 0.0082 to − 0.0005 and − 0.9 to 14.6%, respectively, when the partial view is used and in the range from − 0.0006 to 0.012 and − 21.4 to 0.5%, respectively, for the full view (Table 7). The value of the *MAD* and *MADN* is in the range from 0.0025–0.0046 to 1.8–5.9%, respectively, when the partial view is used and in the range from 0.0017–0.0083 to 2.1–6.2%, respectively, for the full view (Table 7).

**Table 6:** The smallest allowable averaging area (SAAA) of ε_L_ estimates versus the limit of the MaxNE_ES_

Seg	V	SAAA (mm^2^)		
		MaxNE_ES_ < 16.7%	MaxNE_ES_ < 33%	MaxNE_ES_ < 66%
17	F	> 278	278	77
	P	> 278	139	< 3
13	F	< 3	< 3	< 3
	P	< 3	< 3	< 3
7	F	< 3	< 3	< 3
	P	< 3	< 3	< 3
1	F	> 278	< 3	< 3
	P	< 3	< 3	< 3

A2C projection. Notation: V.—View; F—Full; P—Part; <—indicates that SAAA is smaller than the minimal size of the subsegment used because the MaxNE_ES_ error is below the limit even in the case of parcellation on the smallest subsegments used; > —indicates that SAAA is bigger than the maximal size of the AHA segment used because the MaxNE_ES_ error is above the limit

**Table 7:** Error measures of ε_L_ estimates obtained using SAAA and MaxNE_ES_ limit 33%

Seg	V	*MED* [1/1]	*MAD* [1/1]	*MEDN* (%)	*MADN* (%)	*MaxE*_*ES*_ [1/1]	*MaxNE*_*ES*_ (%)	*MXCC* [1]
17	F	0.0120	0.0017	− 21.4	3.0	0.017	30.3	0.99908
	P	− 0.0082	0.0033	14.6	5.9	0.018	32.2	0.99964
13	F	− 0.0006	0.0038	0.5	3.3	0.012	10.2	0.99982
	P	− 0.0021	0.0037	1.7	3.2	0.013	11.4	0.99988
7	F	0.0013	0.0030	− 0.9	2.1	0.010	7.3	0.99995
	P	− 0.0005	0.0025	0.4	1.8	0.008	5.8	0.99994
1	F	0.0155	0.0083	− 11.5	6.2	0.040	30.2	0.99881
	P	− 0.0019	0.0046	1.4	3.6	0.016	12.1	0.99939

A2C projection in selected segments. Seg.—number of AHA17 segment; View—View type; MaxE_ES—_predicted maximal estimation error; MaxNE_ES—_predicted maximal estimation relative error; MXCC—median XCC value; MED—median estimation error; MEDN—median estimation relative error; MAD—median absolute deviation of estimation error; MEDN—median absolute deviation of estimation relative error; V.—View; F—Full; P—Part

The *MaxE*_*ES*_ and *MaxNE*_*ES*_ errors of the *ε*_*R*_ estimate obtained in the A2C projection are in the range 0.025–0.27 and 22–125% respectively (Fig. 12 right). Both error measures tend to decrease as the size of the subsegment increases. The *MaxE*_*ES*_ and *MaxNE*_*ES*_ errors of the *ε*_*R*_ estimates take lower values if partial views are used as compared to the full view results except for the segment no 7, where the opposite is observed. The smallest difference of the *MaxE*_*ES*_ and *MaxNE*_*ES*_ resulting from different view type is observed for the segment no 13. The smallest values of these errors are measured for the AHA17 segment no 17 imaged using the partial view. *MaxE*_*ES*_ in this segment does not exceed 0.075. The *SAAA* is above 278 mm^2^, i.e. the entire area of this segment (Table 8) when the *MaxNE*_*ES*_ limit is 33%, in all segments, except for the segment no 17. The value of *SAAA* obtained in this segment is above 278 mm^2^ for the full view and equals 92 mm^2^ for the partial view, which corresponds to no division or to a division into 3 subsegments, respectively (Table 8). In the case of the *MaxNE*_*ES*_ limit equal 16.7% the *SAAA* exceeded the area of a single AHA17 segment in all cases. The most complex results are observed for *MaxNE*_*ES*_ limit equal 66%. *SAAA* greater than single AHA17 segment area was observed in the segments 13 and 1 regardless of the view type. The *SAAA* values lower than a single AHA17 segment area were observed in other cases. The *SAAA* dropped below 3mm^2^ in the segment 7 when the partial view was used. The value of *MXCC* for *SAAA* obtained in the case of the *MaxNE*_*ES*_ limit equal 33% exceeds 0.965 when the partial view is used and 0.768 when the full view is used (Table 9). For all segments viewed in the A2C projection the values of *MED* and *MEDN* in the case of *ε*_*R*_ are in the range from − 0.097 to − 0.006 and − 51.8% to − 5.4%, respectively, when the partial view is used, and in the range from − 0.116 to − 0.029 and − 63.3% to − 28.1%, respectively, for the full view (Table 9). The values of *MAD* and *MADN* are in the range from 0.006–0.013 to 4.2–6.8%, respectively, when the partial view is used, and in the range from 0.005–0.021 to 3.7–11.2%, respectively, for the full view (Table 9).

**Table 8:** The smallest allowable averaging area (SAAA) of ε_R_ estimates versus the limit of the MaxNE_ES_

Seg	V	SAAA (mm^2^)		
		MaxNE_ES_ < 16.7%	MaxNE_ES_ < 33%	MaxNE_ES_ < 66%
17	F	> 278	> 278	139
	P	> 278	92	92
13	F	> 278	> 278	> 278
	P	> 278	> 278	> 278
7	F	> 278	> 278	< 3
	P	> 278	> 278	139
1	F	> 278	> 278	> 278
	P	> 278	> 278	> 278

A2C projection in selected segments. Notation as in Table 6

**Table 9:** Error measures of ε_R_ estimates obtained for SAAA and MaxNE_ES_ limit 33%

Seg	V	*MED* [1/1]	*MAD* [1/1]	*MEDN* (%)	*MADN* (%)	*MaxE*_*ES*_ [1/1]	*MaxNE*_*ES*_ (%)	*MXCC* [1]
17	F	− 0.029	0.005	− 28.1	5.3	0.045	44.0	0.997
	P	− 0.006	0.006	− 5.4	5.4	0.024	21.5	0.997
13	F	− 0.067	0.016	− 37.5	9.0	0.115	64.5	0.925
	P	− 0.049	0.010	− 27.0	5.6	0.080	43.7	0.994
7	F	− 0.055	0.006	− 32.7	3.7	0.074	43.7	0.882
	P	− 0.090	0.007	− 51.8	4.2	0.112	64.3	0.965
1	F	− 0.116	0.021	− 63.3	11.2	0.178	97.0	0.768
	P	− 0.097	0.013	− 51.6	6.8	0.135	71.8	0.981

A2C projection in selected segments. Notation as in Table 7

**Fig. 13:**
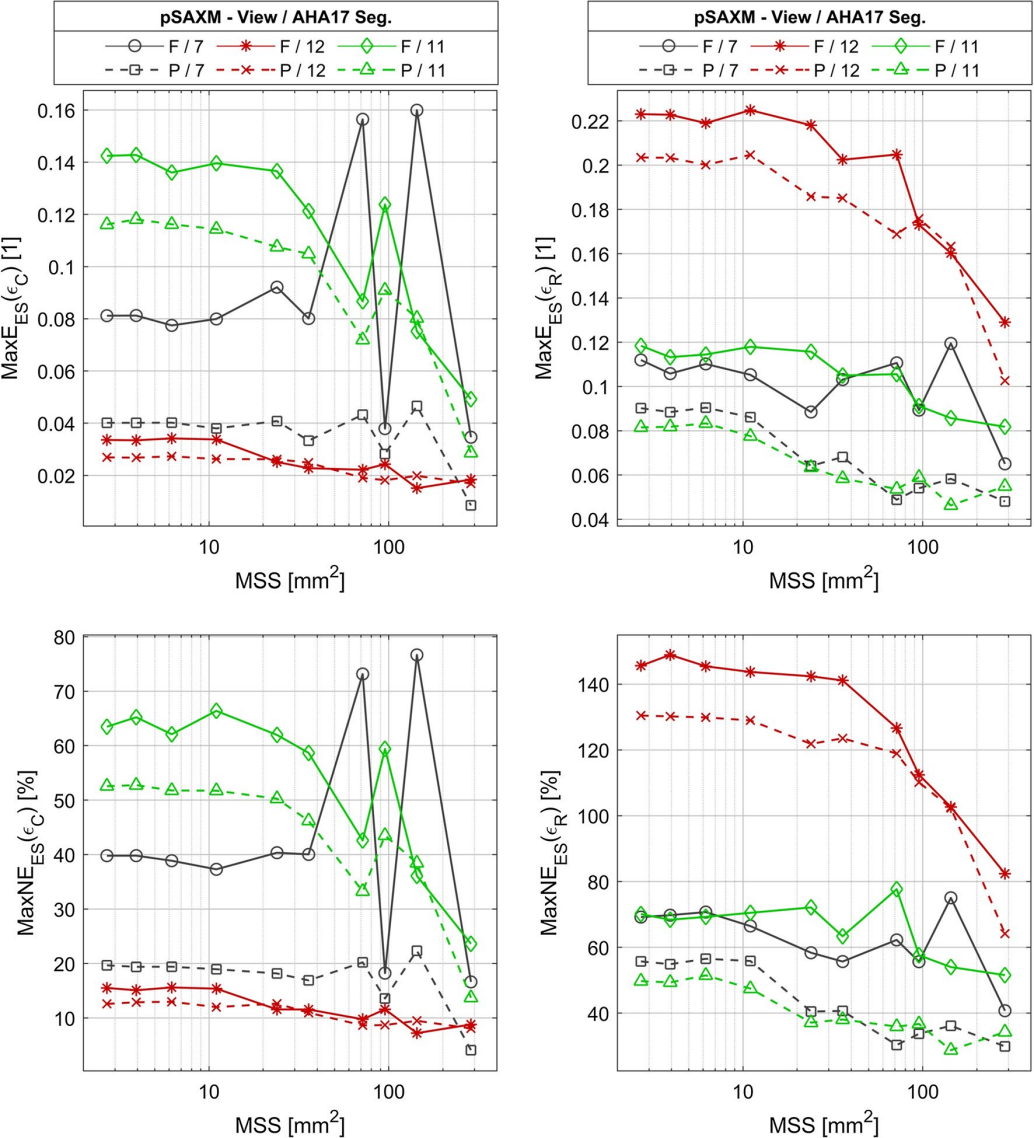
Plots of maximal absolute error (MaxE_ES_) and maximal normalized error (MaxNE_ES_) of circumferential (ε_C_) and radial (ε_R_) as a function of mean surface area of subsegments viewed in the pSAXM projection. *F* full view; *P* partial view. *MSS* mean value of the subsegment surface area

The *MaxE*_*ES*_ and *MaxNE*_*ES*_ errors of the *ε*_*C*_ estimates are in the range 0.009—0.16 and 4.1–77% respectively (Fig. 13 left). Both measures tend to decrease as the size of the subsegment increases, however show unexpected behavior for the 7th segment and for MSS above approximately 50 mm^2^. The *MaxE*_*ES*_ and *MaxNE*_*ES*_ errors of the *ε*_*C*_ estimates in most cases have lower values if the partial view is used as compared to the full view results. The greatest difference between these measures is observed in the AHA17 segment no 7. The lowest error value is observed for the AHA17 segment no 12 imaged using the partial view. The values of *MaxE*_*ES*_ and *MaxNE*_*ES*_ in this case do not exceed 0.027 and 12.6% respectively. The value of error depends on the AHA17 segment position within the imaging sector. In the segment no 12 (laying most laterally in the sector), regardless of the view type, the estimated value of *SAAA* is below 3 mm^2^ for *MaxNE*_*ES*_ limit equal 33% (Table 10). The value of *SAAA* estimated in the segment no 7 (the closest to ultrasonic probe) is 287 mm^2^ for the full view and below 3 mm^2^ for the partial view, which respectively correspond to the entire AHA segment and to the finest division into subsegments (Table 10). In the segment no 11 (most distant from the probe) the estimated value of *SAAA* equals 287 mm^2^ regardless of the view type and corresponds to the entire AHA segment (Table 10). When the smallest (16.7%) *MaxNE*_*ES*_ limit is considered, the *SAAA* smaller than the area of the single AHA17 segment is found only for segment no 12. In the case of the largest *MaxNE*_*ES*_ limit (66%) the *SAAA* only once exceeds 3 mm^2^ in the case of the segment 7 and when the full view is used. The value of *MXCC* obtained for *SAAA* exceeds 0.9991 in all AHA17 segments viewed in the pSAXM projection regardless of the used view type (Table 11). The values of *MED* and *MEDN* for all segments viewed in the pSAXM projection are in the range from − 0.009 to − 0.001 and 0.5 to 4.5%, respectively, when the partial view is used and in the range from − 0.002 to 0.031 and − 15 to 0.9%, respectively, for the full view (Table 11). The value of *MAD* and *MADN* is in the range from 0.006–0.012 to 3.08–5.98%, respectively when the partial view is used and in the range from 0.001–0.011 to 0.53–4.86%, respectively, for the full view (Table 11).

**Table 10:** The smallest allowable averaging area (SAAA) of ε_C_ estimates versus the limit of the MaxNE_ES_

Seg	V	SAAA (mm^2^)		
		MaxNE_ES_ < 16.7%	MaxNE_ES_ < 33%	MaxNE_ES_ < 66%
7	F	287	287	287
	P	287	< 3	< 3
12	F	< 3	< 3	< 3
	P	< 3	< 3	< 3
11	F	> 287	287	< 3
	P	287	287	< 3

SAXM projection in selected segments. Notation as in Table 6

**Table 11:** Error measures of ε_C_ estimates using obtained for SAAA and MaxNE_ES_ limit 33%

Seg	V	*MED* [1/1]	*MAD* [1/1]	*MEDN* (%)	*MADN* (%)	*MaxE*_*ES*_ [1/1]	*MaxNE*_*ES*_ (%)	*MXCC* [1]
7	F	0.031	0.001	− 15.0	0.53	0.03	16.6	0.9997
	P	− 0.004	0.012	1.7	5.98	0.04	19.7	0.9994
12	F	− 0.002	0.011	0.9	4.86	0.03	15.5	0.9991
	P	− 0.001	0.009	0.5	4.03	0.03	12.6	0.9992
11	F	0.023	0.009	− 11.0	4.21	0.05	23.6	0.9994
	P	− 0.009	0.006	4.5	3.08	0.03	13.7	0.9996

pSAXM projection in selected segments. Notation as in Table 7

The *MaxE*_*ES*_ and *MaxNE*_*ES*_ error of radial strain (*ε*_*R*_) estimates obtained in the pSAXM projection are in range from 0.046 to 0.23 and from 29 to 149% respectively (Fig. 13 right). Both errors tend to decrease as the size of subsegment increases. The *MaxE*_*ES*_ and *MaxNE*_*ES*_ errors of *ε*_*R*_ estimates have lower values if the partial view is used as compared to the full view results. The difference of the *MaxE*_*ES*_ and *MaxNE*_*ES*_ obtained for different view types is observed in the segment no 11. The smallest values of errors are measured for AHA17 segment no 11 imaged using the partial view. The values of *MaxE*_*ES*_ and *MaxNE*_*ES*_ in this case do not exceed 0.082 and 50% respectively. The *SAAA* value for all segments is above or equal to 287 mm^2^ and corresponds to no segment division regardless of the view type (Table 12) when the *MaxNE*_*ES*_ limit is less than or equal to 33%. For the largest limit of the *MaxNE*_*ES*_ (66%) the *SAAA* is smaller than the area of the entire AHA17 segment in three cases, i.e. for segment 7 when full view is used and for segment 11 independently of the view type. The *MXCC* always exceeds 0.884 when the partial view is used and 0.698 when the full view is used (Table 13). For all segments viewed in the pSAXM projection the *ε*_*R*_ value of *MED* and *MEDN* is in the range from − 0.088 to − 0.021 and − 55.3 to − 13.2% when the partial view is used and in the range from − 0.085 to − 0.049 and − 54.2 to 30.9% for the full view (Table 13). The value of *MAD* and *MADN* is in the range from 0.005–0.011 to 2.9–7% when the partial view is used and in the range from 0.005 to 0.015 and − 3.3 to 9.4% for the full view (Table 13).

**Table 12:** The smallest allowable averaging area (SAAA) of ε_R_ estimates versus the limit of the MaxNE_ES_

Seg	V	SAAA (mm^2^)		
		MaxNE_ES_ < 16.7%	MaxNE_ES_ < 33%	MaxNE_ES_ < 66%
7	F	> 287	> 287	287
	P	> 287	287	< 3
12	F	> 287	> 287	> 287
	P	> 287	> 287	> 287
11	F	> 287	> 287	95
	P	> 287	> 287	< 3

SAXM projection in selected segments. Notation as in Table 6

**Table 13:** Error measures of ε_R_ estimates obtained for SAAA and MaxNE_ES_ limit 33%

Seg	V	*MED* [1/1]	*MAD* [1/1]	*MEDN* (%)	*MADN* (%)	*MaxE*_*ES*_ [1/1]	*MaxNE*_*ES*_ (%)	*MXCC* [1]
7	F	− 0.049	0.005	− 30.9	3.3	0.07	40.7	0.988
	P	− 0.032	0.005	− 20.0	3.3	0.05	29.9	0.994
12	F	− 0.085	0.015	− 54.2	9.4	0.13	82.4	0.698
	P	− 0.088	0.005	− 55.3	2.9	0.10	64.2	0.884
11	F	− 0.052	0.010	− 33.0	6.2	0.08	51.6	0.980
	P	− 0.021	0.011	− 13.2	7.0	0.05	34.3	0.996

pSAXM projection in selected segments. Notation as in Table 7

The plots of the error measures frequently show a change of the slope (Figs. 12, 13) e.g. the plot of *MaxE*_*ES*_(*ε*_*R*_) for segment no 12. The errors usually start to drop faster when the *MSS* values reach the range of 20 mm^2^ to 100 mm^2^.

## Discussion

The error of the strain components estimates strongly varies as does the smallest allowable averaging area of the estimation. The highest accuracy is observed for the longitudinal strain estimates. Using the A2C projection, the *ε*_*L*_ could be estimated in the model using much smaller averaging area than that currently clinically used. This applies to the entire LV model wall even if the *MaxNE*_*ES*_ limit equals 16.7%, except for the apex. The close to 1 cross-correlation coefficient between the *ε*_*L*_ reference plot and the corresponding estimate indicates significant synchronism and correlation of both, even for a small averaging area, i.e. high resolution. This suggests that 2DSTE estimates of *ε*_*L*_ can be used to evaluate local changes in the LV model wall. A significant influence of image acquisition parameters is observed at the apex (segment no 17) and at the base (segment no 1), where the use of the partial view leads to the estimation error reduction and (at the apex) allows to use a small averaging area, i.e. to increase the spatial resolution of estimation.

The dimension of the smallest detectable infarct using longitudinal strain reported in [6] was 1.9 cm. The simulated lesion covered the entire wall thickness, so the overall area appearing in the A2C view was approximately 190 mm^2^, which is close to the largest subsegment area used here. It is important to note, that the 1.9 cm size of the area was also the smallest examined case in the cited study. The results of our study suggest that the smallest detectable infarct could be smaller than reported in [6].

The results indicate that the errors in the case of the circumferential strain (*ε*_*C*_) estimation are strongly related to the position of the region of interest (ROI) in the image sector and the parameters of the image acquisition. The SAAA is sensitive to the *MaxNE*_*ES*_ limit in majority of cases.

In the lateral segments (e.g. segment no 12) the SAAA could be much smaller than the size of the clinically used segments (AHA17) regardless of view type while keeping the *MaxNE*_*ES*_ low. In the case of other segments using a averaging area smaller than a single AHA segment is possible only when the allowable maximal error is 66% or higher.

When the ROI is located close to the ultrasonic probe (e.g. segment no 7) the strongest impact of the view type is observed. Using a partial view may lead to estimation error reduction and allows to significantly reduce (100-fold) the strain averaging area as compared to those seen when the full view was used. This high sensitivity to the view type may result from different image line density and therefore different lateral resolution of image data in both cases. An unexpected behavior of the error measures, i.e. high difference between subsequent values was observed for the 7th segment imaged using the full view. This phenomenon could result from the relatively small number of data samples and properties of the error measures. We conjecture that in the case when the strain estimation error is relatively high and arises from a small area, this area may fall into one or more subsegments at different stages of the division into subsegments. The median value of error may strongly vary depending on the number of subsegments affected by this error, in particular when the number of subsegments submitted to the median error estimation is low.

In the most distant regions (e.g. segment no 11) the error of *ε*_*C*_ estimate is relatively high regardless of the view type. This limits the averaging area to the typically used gross segments (like AHA17). Taking into account the low SAAA (high resolution) found for close regions, achieving this situation (high resolution) in the case of *ε*_*C*_ in segments distant in the transthoracic approach may require using another imaging strategy, e.g. transesophageal echocardiography.

The highest estimation errors are seen in the case of the radial strain estimates (*ε*_*R*_) regardless of the projection. This is in accordance with the common opinion on superiority of longitudinal strain estimate component as compared to radial strain estimate. The poorer quality and reproducibility of radial and circumferential strain estimates are also the problem in the commercial software and it is reported by clinicians [65]. The strain estimation error limit of 33% is reached already for gross segments (like AHA17) in all but one cases, regardless of acquisition settings (full or partial view). The SAAA is lower than the entire AHA17 segment in almost half of the cases when the *MaxNE*_*ES*_ limit equals 66%. This indicates that that even for this strain component it is possible to increase the resolution of strain estimation if requirements for accuracy are not high.

The changes of the slope of the *MaxE*_*ES*_ and *MaxNE*_*ES*_ (Figs. 12, 13) indicate that the estimation errors as a function of the averaging area grow only to some point behind which the rate of error change is significantly reduced. This could stem from the spatial resolution of displacement and also from the fact, that the strain estimation procedure comprises low pass filtering that limits rapid change of the estimation error. Averaging over a smaller subsegment size than that of the filter implemented in strain estimation could be considered as the use of another low pass filter having larger bandwidth than the first one. The impact of the second filter is thus limited and results are mostly related to the bandwidth of the first filtering.

Strain estimation described here is carried out using a block-matching algorithm and thus this work evaluates the performance of a particular method. Such methods serve also as a basis for model based approaches where many more information sources are used to reach the desired diagnostic conclusions. Such work presented in [66] has shown the ability of a model-based strain estimation approach to identify an infarction with a volume as low as 5 ml, but this has been achieved using both MR and echocardiographic data to tune individualized electromechanical ventricle models and to label mesh elements as infarcted or not.

This study has some limitations. The LV model proposed is simple, however enables fast and easy implementation and ensures satisfactory similarity of the model shape, size and deformation parameters to the clinical data. The study is limited to one 2DSTE method (i.e. HBM) and synthetic data. Field II is a linear simulation program and does not support simulation of many artifacts seen in vivo. Both imply simplification of image acquisition and some sacrifice of data realism. However, using this simplified synthetic data enables setting acquisition parameters like projection planes, number of lines and frame rate (Table 2) which are usually fixed in publicly available synthetic echo image databases [29]. Furthermore, synthetic data based on a FEM model is provided with accurate deformation reference data with high spatial resolution. Such a reference is crucial for the experiment described here and is not available in clinic nor in physical models. Running a similar study using a more realistic dataset, as the one published in [29] could provide interesting results, complementary to the outcome of the work reported here. This new study could be extended over the vendor-specific imaging conditions, but would not include all the variables taken into account here. Another complementary study could address the comparison of commercial algorithms and the one used here, however in such a case the objective assessment of errors may not be possible, as the ground truth data would not be available.

## Conclusions

We have presented a methodology and the results of evaluation of the tradeoff between strain averaging area and strain estimation errors in a spheroidal left ventricular model. We proposed the smallest allowable averaging area (SAAA) of this estimation that will keep the strain estimation error below the proposed limit that could be clinically acceptable.

The errors of the estimation of individual strain components vary strongly as does the *SAAA*. The highest accuracy is observed in the longitudinal strain estimates when the partial view is used. In all segments but 17th in the A2C view the *ε*_*L*_ could be estimated using the averaging area below 3 mm^2^ (thus high spatial resolution), still keeping the estimation error below suggested here admissible level, i.e. still enabling the distinction between the viable and the infarcted tissue. This resolution is far better than the resolution corresponding to an AHA segment currently used in clinical settings. 2DSTE estimates of *ε*_*L*_ could then be used to evaluate the local condition of the LV muscle and may have a potential for diagnosis of such pathologies as a non-transmural infarct. The circumferential strain (*ε*_*C*_) estimation errors strongly depend on the position of the ROI in the image sector and the parameters of the image acquisition. The radial strain estimates (*ε*_*R*_) show the highest median of relative errors. This emphasizes the inferior clinical utility of the radial strain as compared to the circumferential and longitudinal ones [65]. The results presented here suggests also that the use of a narrow sector (local imaging) could increase diagnostic capabilities of 2DSTE.

The close to 1 values of *MXCC* obtained for estimates of *ε*_*L*_ and *ε*_*C*_ and the corresponding reference waveforms suggest high correlation and synchronism. This suggests that 2DSTE estimates of *ε*_*L*_ and *ε*_*C*_ could be used to evaluate the local dyssynchrony of the LV.

According to the best knowledge of the Authors, this study is the first attempt of a systematic and quantitative assessment of the smallest allowable averaging area (SAAA) of strain estimation in a LV model. The SAAA may be expected to ensure clinically acceptable accuracy together with improved resolution when using 2DSTE method. The results may suggest that potential of the 2DSTE as diagnostic tool may not be currently fully utilized in clinic.

## Data Availability

The datasets and code used and analyzed during the current study are available from the corresponding author on reasonable request.
